# Supplementary material for: Spatial Variation in Stream-Water Bacterial Communities Across Sites Differing in Wild Chinese Giant Salamander Detection Frequency
Source: Biology (Basel). 2026 Sep 18;15(18):1647. doi: 10.3390/biology15181647 (PMC13604818; doi:10.3390/biology15181647)
Supplement: Supplementary file 1 [file biology-15-01647-s001.zip › Data Related to “Spatial Variation in Stream-Water Bacterial Communities across Sites Differing in Wild Chinese Giant Salamander Detection Frequency”/Microbial Data/result/03.TaxonVisual/heatmap/sample/heatmap.sample.f.pdf]

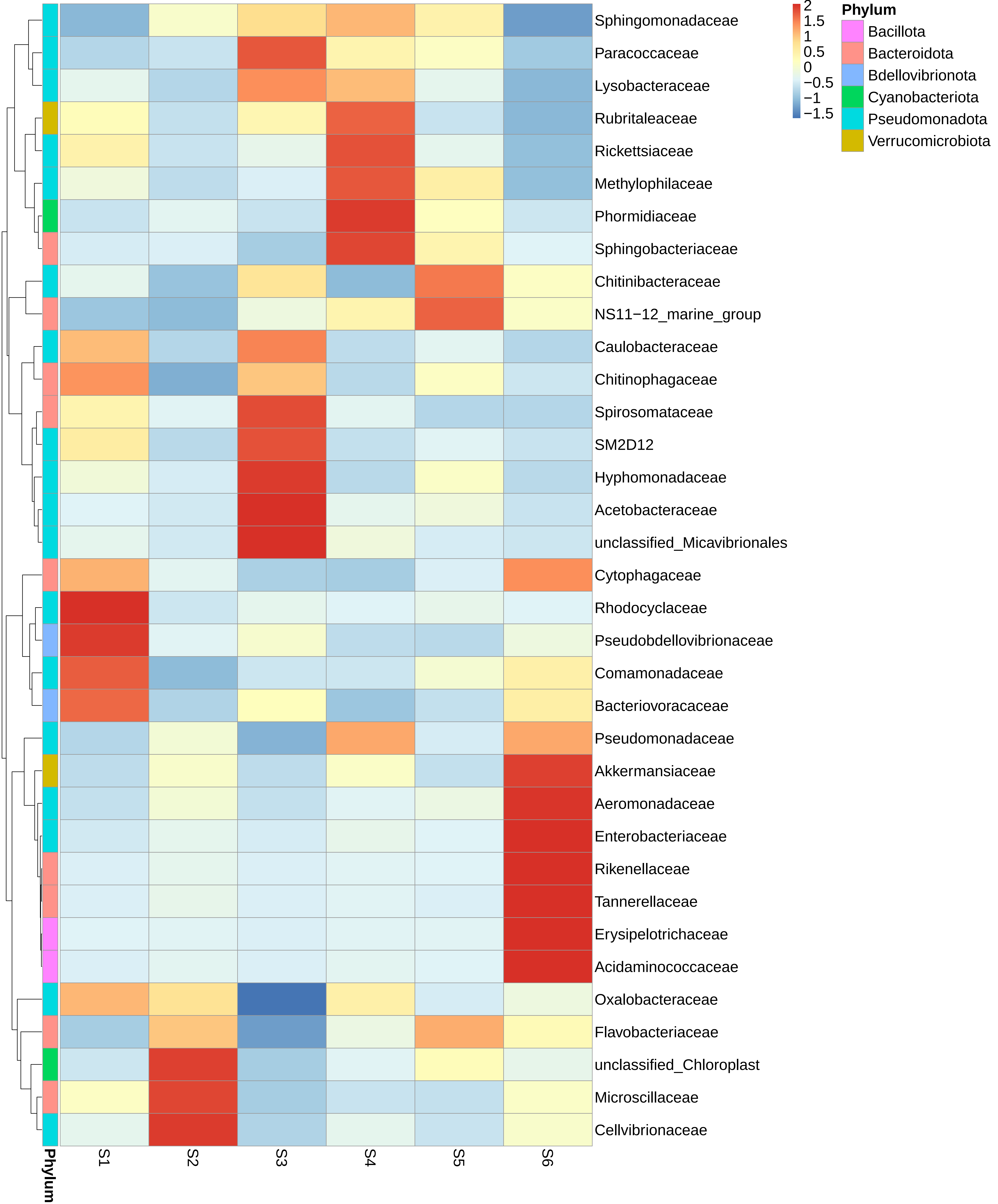
Sphingomonadaceae
Paracoccaceae
Lysobacteraceae
Rubritaleaceae
Rickettsiaceae
Methylophilaceae
Phormidiaceae
Sphingobacteriaceae
Chitinibacteraceae
NS11−12_marine_group
Caulobacteraceae
Chitinophagaceae
Spirosomataceae
SM2D12
Hyphomonadaceae
Acetobacteraceae
unclassified_Micavibrionales
Cytophagaceae
Rhodocyclaceae
Pseudobdellovibrionaceae
Comamonadaceae
Bacteriovoracaceae
Pseudomonadaceae
Akkermansiaceae
Aeromonadaceae
Enterobacteriaceae
Rikenellaceae
Tannerellaceae
Erysipelotrichaceae
Acidaminococcaceae
Oxalobacteraceae
Flavobacteriaceae
unclassified_Chloroplast
Microscillaceae
Cellvibrionaceae
S1
S2
S3
S4
S5
S6
Phylum
2
1.5
1
0.5
0
−0.5
−1
−1.5
Phylum
Bacillota
Bacteroidota
Bdellovibrionota
Cyanobacteriota
Pseudomonadota
Verrucomicrobiota
